# Understanding Childhood Fever: A Qualitative Study of Family Attitudes, Emotions, and Caregiving Practices

**DOI:** 10.3390/children12121584

**Published:** 2025-11-21

**Authors:** Francisco Vicens-Blanes, Jesús Molina-Mula, Rosa Miró-Bonet

**Affiliations:** Department of Nursing and Physiotherapy, Balearic Islands University, Carretera de Valldemossa, km 7.5, 07122 Palma, Spain; jesus.molina@uib.es (J.M.-M.); rosa.miro@uib.es (R.M.-B.)

**Keywords:** parents, children, fever

## Abstract

**Highlights:**

**What are the main findings?**

- Mothers view childhood fever as a disruptive event requiring rapid decisions, shaped by emotions, prior experiences, culturally transmitted knowledge, and social context.
- Routine use of antipyretics, physical remedies, and early emergency consultations are common; the caregiver–professional relationship is ambivalent, highlighting the need for better health education and greater emotional support.

**What are the implications of the main findings?**

- Guides pediatric and emergency clinicians in using tools to provide more empathetic, culturally sensitive care for childhood fever.
- Helps families by improving communication about childhood fever care, reducing caregiver anxiety, and enhancing the caregiving experience—especially in Spanish-speaking contexts.

**Abstract:**

**Introduction:** Although childhood fever is a common and typically self-limiting clinical presentation, it imposes a substantial emotional and psychological burden on caregivers, especially mothers, primarily due to enduring practices and beliefs that diverge from contemporary evidence-based medical guidelines. **Objective:** The aim was to investigate how families perceive pediatric fever, identify the factors influencing their caregiving decisions, and examine their experiences across various healthcare settings. **Methodology:** A qualitative study employing an ethnomethodological approach was conducted at a tertiary care hospital in Spain. The study included ten caregivers (nine mothers and one father) of children presenting with fever. Data were collected through in-depth interviews and field diary observations. Analysis was performed using a combination of inductive and deductive methods, supported by the Atlas.ti 25.0.1 version software. **Results:** Mothers perceive childhood fever as a disruptive event necessitating rapid decision-making, shaped by emotional responses, prior experiences, culturally transmitted knowledge, and social context. Common caregiving practices included routine administration of antipyretics, application of physical remedies, and early utilization of emergency healthcare services. The caregiver–healthcare professional relationship was characterized by ambivalence, highlighting the need for enhanced health education and greater emotional support for caregivers. **Conclusions:** Childhood fever represents both a clinical condition and a sociocultural phenomenon. Gaining insight into family practices and beliefs enables the development of more effective, empathetic educational interventions, which can enhance caregiving practices and mitigate parental anxiety. Reporting method: Standards for Reporting Qualitative Research (SRQR) guidelines.

## 1. Introduction

Fever is a common and regulated physiological response to infection, characterized by an increase in core body temperature beyond the normal circadian range [1,2]. This increase results from the release of endogenous pyrogens that alter the hypothalamic thermoregulatory set point, creating a hostile environment for pathogenic microorganisms [3]. In contrast to hyperthermia, fever typically does not exceed 41 °C and is a controlled, non-lethal process [3,4].

Fever is one of the most frequent clinical signs during infancy, particularly between the ages of 3 and 36 months, a period in which children commonly experience two to six febrile episodes per year. These episodes are typically mild and of viral origin [1,4,5,6,7,8]. In infants younger than three months, however, any febrile episode is of particular clinical concern due to an increased risk of serious infections, related to immune system immaturity and the nonspecific nature of clinical symptoms [1,5,9,10,11].

Scientific literature consistently emphasizes that the clinical risk associated with fever lies primarily in its underlying etiology rather than the elevation in body temperature itself. As a result, fever typically causes only transient discomfort and, in most cases, does not warrant specific medical intervention [1,5,10,11]. Consequently, organizations such as the National Institute for Health and Care Excellence [12] recommend that the management of fever in children should not be primarily directed at reducing body temperature, but rather at alleviating discomfort and ensuring adequate hydration. This evidence-based approach necessitates a comprehensive clinical assessment that incorporates factors such as the overall condition, level of distress, and age, rather than relying completely on temperature measurements [10].

Fever is among the most common causes of pediatric consultations in healthcare settings, including both primary care and emergency departments [13,14,15]. Within this context, healthcare professionals play a critical role in providing guidance and education to parents, helping them understand appropriate fever management and when to seek medical attention. However, empirical evidence suggests that healthcare professionals may not always possess a consistent or evidence-based understanding of fever, which can lead to the dissemination of contradictory or inaccurate information to caregivers [1,16]. From the perspective of social learning theory [17], this issue is particularly significant, given the influential role that healthcare providers serve as behavioral models for parents and caregivers in managing childhood illness. Despite the availability of clinical guidelines and the accumulation of evidence-based knowledge, a persistent gap remains between professional recommendations and the fever management practices adopted by families. Parents often perceive fever as a dangerous and urgent medical condition, even though, in most cases, it is a benign physiological response [1,16,18]. This misperception contributes to the phenomenon known as “feverphobia,” an emotionally driven response characterized by increased anxiety and an exaggerated fear of mild febrile episodes [3,19].

Moreover, research has shown that certain unfounded fears, such as concerns about fever-induced brain damage, death, or an increased risk of seizures, are prevalent not only among parents but also among healthcare professionals, despite their weak scientific support [20,21,22,23,24,25,26,27]. These misconceptions contribute to the persistence of inappropriate fever management practices and maintain the misguided belief that fever is inherently harmful.

In this context, numerous studies have emphasized the importance of understanding caregivers’ beliefs, attitudes, and lived experiences as a means to enhance the quality of pediatric health care and to better tailor professional interventions to the specific needs and expectations of Families [2,7,11,14,28,29]. Parental perceptions of fever are shaped by a complex interaction of cultural, social, and educational factors, all of which influence their health-related decision-making and behaviors [30].

Maternal concern tends to be especially pronounced in cases where there is a history of febrile seizures. Although these events are generally associated with a favorable clinical prognosis, they are often perceived as traumatic by caregivers and can lead to significant behavioral changes. In particular, parents may become hypervigilant in monitoring body temperature and more likely to administer antipyretic medications preemptively or more frequently [31,32,33].

Ongoing distress among families, coupled with insufficient or unclear health education, contributes to the persistence of inappropriate practices in fever management. A recent systematic review found that, although many parents do not inherently view fever as dangerous, they nonetheless interpret it as a condition requiring immediate intervention, often prioritizing the reduction in body temperature as the primary objective of care [34].

To investigate this phenomenon comprehensively from the familial perspective, qualitative research methodologies are most appropriate. Specifically, ethnomethodology provides a robust framework for examining how individuals perceive, interpret, and structure their everyday practices, making it particularly well-suited for analyzing the domestic management of pediatric fever [35,36]. In light of these considerations, the present study aims to elucidate families’ perceptions, knowledge, and attitudes regarding childhood fever, as well as their experiences across diverse healthcare settings.

## 2. Materials and Methods

### 2.1. Design

This study employs a qualitative design within the constructivist paradigm. An ethnomethodological approach is adopted to analyze families’ perceptions, knowledge, and attitudes toward childhood fever, with a focus on understanding how these are constructed and enacted in everyday contexts.

An ethnomethodological approach is particularly well-suited for qualitative research, as it seeks to examine how members of a social group perceive, interpret, and categorize routine activities, along with the meanings they attribute to them. This methodology focuses on aspects of social life that are often taken for granted, practices so embedded in daily routines that they require deliberate reflection to disclose their underlying significance and contextual value [35,36].

In operational terms, this philosophical approach was first translated into data collection, guiding the techniques used (described in Section 2.4) so that families were encouraged to recount real situations of fever in their children and the everyday activities they perform to manage it. Mothers and fathers were encouraged to use their own language and categories to describe what they consider to be a fever, when they become concerned, which interventions they initiate, and how they evaluate their effectiveness.

Ethnomethodology guided the data analysis, which focused on the ways in which families perceive, define, and classify fever and its care. From the conversations, we identified the expressions, classifications, and arguments used by families to make sense of childhood fever, and we examined the meanings they attribute to practices that are usually taken as “normal” or “common sense”. In this way, the ethnomethodological approach not only provides the theoretical framework for the study but also explicitly informs both how information is obtained and how it is interpreted.

### 2.2. Theoretical Perspective: Reflexivity and Positioning

A theoretically pluralistic approach guides the presentation and interpretation of the study’s findings. Harper’s framework is used to articulate the fundamental concepts of ethnomethodology and to situate them within the context of the phenomenon under investigation [36]. In addition, in order to achieve a level of interpretation that goes beyond a simple description of the data, elements from the work of three authors are incorporated: Bolívar, Freire, and Bandura [17,37,38].

The contributions of Antonio Bolívar [37] have been useful for understanding attitudes within his frame of reference and for examining how professional roles and context influence the management of childhood fever, as well as how these dynamics affect families’ attitudes. His proposals regarding conceptual, procedural, and attitudinal contents, together with the notion of a “hidden curriculum,” allow us to analyse not only how values, norms, and dispositions present in healthcare settings shape professional practice, but also how these values are transmitted directly or indirectly to mothers and fathers, influencing the way they perceive and manage their children’s fever.

The concepts developed by Paulo Freire [38] on “banking education” and problem-posing education, in Pedagogy of the Oppressed, are used to analyse the types of knowledge about fever that professionals and families handle, and how such knowledge is constructed and transmitted. This framework is particularly useful for interpreting the experiences of mothers and fathers as recipients of health education: it allows us to distinguish between more unidirectional, information-centred models of communication and more dialogical models that foster critical reflection and a more meaningful understanding of childhood fever and its care.

Finally, the forms of learning and behaviour described by Albert Bandura [17] in his social learning theory make it possible to discuss the transmission of knowledge and practices among professionals, from professionals to families, and also among families themselves. Processes of observing behavioural models, vicarious learning, and the role of self-efficacy provide a framework for understanding how mothers and fathers learn to manage childhood fever by observing what others (professionals, relatives, or other caregivers) do, interpreting the consequences of these behaviours, and reshaping their own caregiving practices.

Taken together, the pedagogical concepts of Bolívar and Freire enable an analysis and understanding of the attitudes and knowledge of mothers and fathers in relation to childhood fever, while Bandura’s theory, from the field of psychology, frames the learning processes and helps to explain how particular practices are acquired and reproduced within families and healthcare contexts. These frameworks are employed as complementary lenses to deepen the interpretation of the discourses and practices identified through the ethnomethodological approach, thereby enriching the discussion of the findings without contradicting the primary framework of the study. The principal investigator is a paediatric nursing specialist with professional and teaching experience in paediatric care, which facilitates the articulation of these theoretical perspectives with the clinical and educational realities experienced by the participating families.

### 2.3. Participants

The study was conducted in the pediatric inpatient unit at a tertiary-level healthcare facility, specifically, the Hospital Universitario Son Llàtzer, located in Palma de Mallorca, Spain. The hospital’s catchment area serves an estimated population of approximately 270,276 individuals, of whom 15.61%, around 42,190, are children between 0 and 14 years of age. The hospital provides specialized pediatric care to this population through a network of 14 affiliated primary healthcare centers within its designated service area. A total of 10 parents, 9 mothers and 1 father, who expressed interest in participating, were enrolled for this study. Participants were identified and contacted through a key informant, the supervisor of the pediatric inpatient unit. Following initial contact, the principal investigator visited the unit to conduct the interviews.

Interviews were conducted either in the hospital room where the child was admitted or in a designated private space within the same pediatric unit, depending on the preferences and comfort of the participants. The data collection period spanned from 29 May 2024, when the first interview was conducted, to 12 July 2024, when the final interview took place.

The inclusion criteria for participant selection were as follows:

- Mothers, fathers, or primary caregivers of a child aged between 3 months and 14 years who were hospitalized in the pediatric inpatient unit due to a fever-related episode at the time of recruitment.
- Mothers, fathers, or primary caregivers who had prior experience managing their child’s fever in various healthcare contexts, including primary care, hospital emergency departments, and inpatient settings.

The exclusion criteria for participants were as follows:

- Parent or primary caregivers who experience significant difficulties in comprehending the interview questions or formulating their responses due to language barriers or other factors.

Families were contacted as the child approached hospital discharge to minimize the potential influence of acute anxiety or concern regarding the child’s medical condition on their responses. Inclusion of parents with experience across the three healthcare settings, primary care, emergency department, and inpatient unit, facilitated exploration of the differing perceptions across levels of care and their impact on caregiver behaviors. Parents of children older than three months were selected due to the clinical significance of fever as a warning sign in this age group. Although both fathers and mothers were invited to participate in the study, the sample predominantly consisted of mothers, with only one father agreeing to be interviewed.

### 2.4. Data Collection

Data were collected using two main methods: in depth interviews and the principal investigator’s field diary. First, a conversational approach was adopted through in-depth interviews guided by open-ended questions. Each interview lasted approximately 30 min. The interview guide is provided in the Supplementary Material accompanying this article.

At the beginning of each interview, sociodemographic data were collected from participants, including age, sex, country of origin, number of children, educational level, and perceived social support (Table 1). Perceived support was recorded and classified as low, medium, or high, which will make it possible to analyse whether the perception of support may modify how febrile episodes are perceived or the attitude towards them.

In addition, clinical and demographic information about the hospitalised child was recorded, including age, sex, presence of chronic conditions, reason for admission, and history of febrile seizures (Table 2). The presence of chronic conditions was documented in order to have this information available when interpreting families’ accounts, as certain conditions may shape or modify the subjective experience of fever and the concerns associated with febrile episodes.

Furthermore, it was recorded whether the child had previously experienced febrile seizures, as this is an event closely related to childhood fever and, according to the literature, has a considerable impact on how both families and professionals respond. In cases where the response was affirmative, an additional set of questions specifically addressing these convulsive episodes was included.

In this way, Table 1 and Table 2 not only describe the sociodemographic and clinical characteristics of the sample, but also provide relevant information for interpreting mothers’ and fathers’ accounts in light of their life context, their support network, and their previous experiences with fever and febrile seizures.

In parallel, the principal investigator kept a field diary throughout the study. This diary included written notes and audio recordings made before and after the interviews, in which observations and reflections (thoughts, emotions, and ideas) were documented, contributing to the enhancement of both the external and internal validity of the research [39]. Based on the observations recorded in the field diary, some interview questions were revised, and new questions were incorporated to address emerging topics identified during preliminary analysis that were relevant to the phenomenon under study.

### 2.5. Data Analysis

The data analysis was based on a discourse-oriented perspective, in which discourse is understood as the use of language as a form of social practice. This perspective makes it possible to examine the meanings constructed in participants’ communication and to interpret these meanings within the broader context of social and cultural norms [40]. From this starting point, the analysis focused on how mothers and fathers talked about childhood fever and its everyday management, paying attention to the words, expressions, and ways in which they described, explained, and classified fever and the interventions they performed.

Data analysis followed a cyclical process of inductive and deductive analysis, organised in a sequence of several phases [40]. First, the data to be analysed were obtained through in-depth interviews and the principal investigator’s field diary notes. Interview data were captured using an audio recorder and subsequently transcribed using the online tool “Sonix” (Sonix Inc., San Francisco, CA, USA). Each transcript was reviewed by the principal investigator to ensure accuracy and completeness.

Second, the resulting texts were anonymised and assigned an acronym according to whether the participant was a mother, father, or other primary caregiver, together with a number for each interview conducted. The texts were then imported into the Atlas.ti 25.0.1 version (ATLAS.ti Scientific Software Development GmbH, Berlin, Germany) program, an advanced software package for qualitative data analysis designed to organise, code, and visualise information in qualitative research. With the support of this software, the transcripts were read in detail, and preliminary underlining and annotations were made to highlight those passages considered most relevant.

Based on the transcribed texts, an inductive analysis was carried out. Interview and observational data were coded systematically, identifying keywords, phrases, or entire paragraphs in order to identify specific themes and determine what was meaningful in relation to families’ knowledge, perceptions, and attitudes toward childhood fever and its management. At this point, the discursive orientation of the study was reflected in the close attention paid to the expressions used by mothers and fathers to name fever, assess its severity, justify interventions, and describe their everyday experience.

Subsequently, a deductive analysis was conducted, which made it possible to examine how the data behaved and to assign meaning to the categories in light of the ethnomethodological approach [40]. Within this cyclical process, an initial descriptive phase of the data and codes provided the basis for a subsequent, more interpretive phase. Finally, the data were grouped into categories and subcategories, integrating the information and extracting meanings, comparisons, and conclusions from an ethnomethodological perspective.

**Figure 1:**
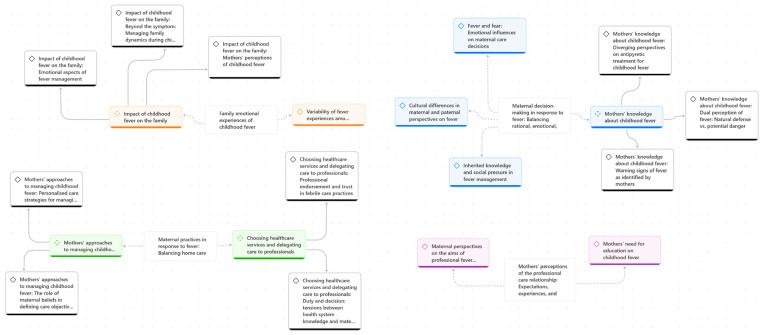
Code network.

**Figure 2:**
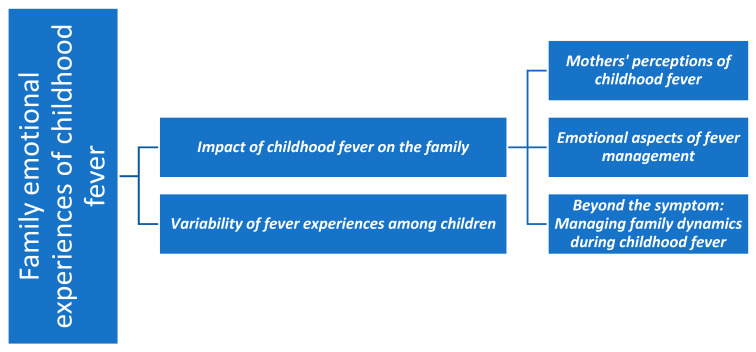
Category: Family emotional experiences of childhood fever.

**Figure 3:**
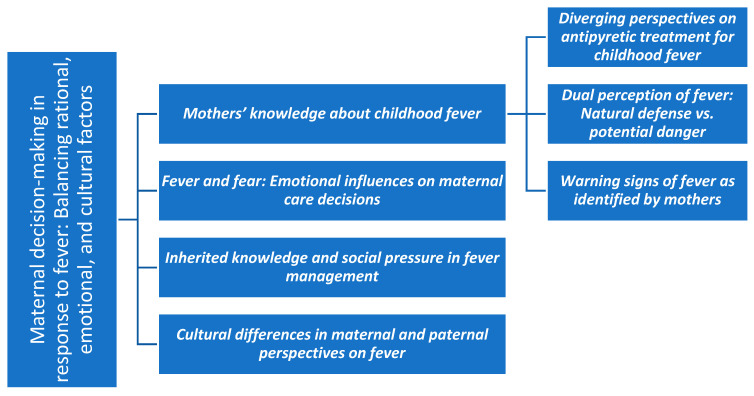
Category: Maternal decision-making in response to fever: Balancing rational, emotional, and cultural factors.

**Figure 4:**
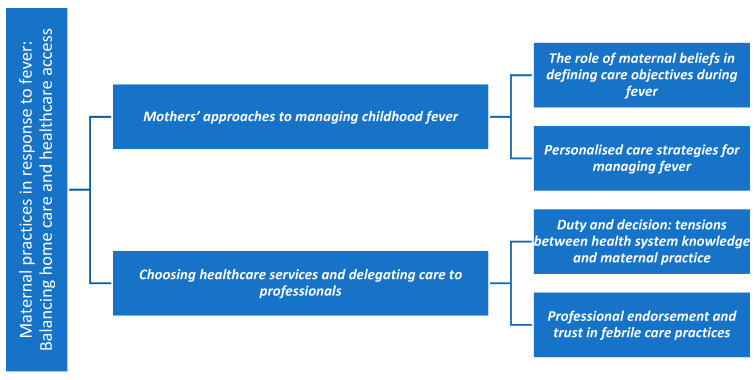
Category: Maternal practices in response to fever: Balancing home care and healthcare access.

**Figure 5:**
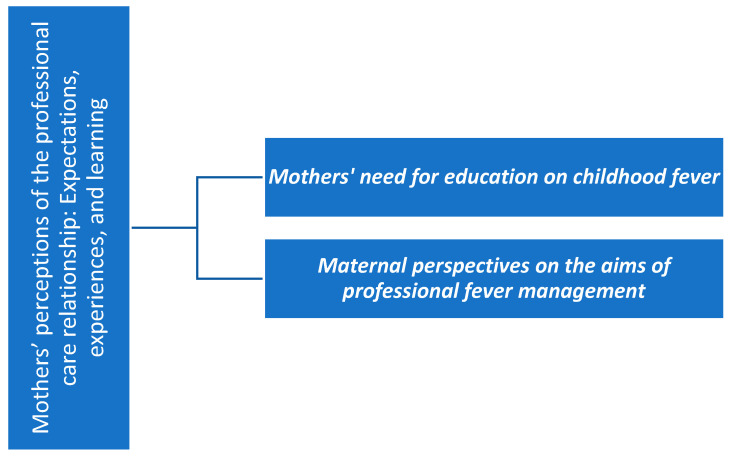
Category: Mothers’ perceptions of the professional care relationship: Expectations, experiences, and learning.

The data were managed, coded, and analysed using the Atlas.ti program (2025). This software also enabled graphical visualisation of the relationships between codes through the development of a code network, which is presented in the Section 3 (Figure 1). In addition, coding trees were manually produced to visually represent the categories, subcategories, and codes analysed; these are presented in Figure 2, Figure 3, Figure 4 and Figure 5.

### 2.6. Strategies for Ensuring Methodological Rigor

Methodological rigor in this qualitative study was maintained through strategies including data triangulation, credibility, and saturation. Triangulation was achieved by involving multiple researchers in the joint analysis of interview data, which enhanced the consistency and reliability of interpretation [41]. Credibility was enhanced through member validation, whereby participants reviewed and confirmed the accuracy of the findings, and by triangulating results with existing literature, while critically reflecting on the researcher’s potential influence on data interpretation [42]. Theoretical saturation was employed as the criterion for concluding data collection, defined as the point at which no new substantive categories or themes emerged.

### 2.7. Ethical Considerations

Participating parents were provided with comprehensive information about the study both verbally and via a written information sheet. Informed consent was obtained through the reading and signing of a consent form by each participant prior to the interviews. All collected data and participants’ information were anonymized and securely maintained by the principal investigator to ensure confidentiality. This study received ethical approval from the Research Ethics Committee of the Balearic Islands (approval reference IB4627/21) on 5 November 2021, as well as from the ethics committee of the Hospital Universitario Son Llàtzer. The research is conducted within the framework of the project titled: ‘Analysis of the knowledge and Attitudes of Professionals and Families towards Childhood Fever. This study has received funding from the Official Association of Nurses of the Balearic Islands (COIBA).

## 3. Results

This section presents the study’s findings, organized into thematic categories and illustrated with selected excerpts from participants’ narratives. Before introducing the categories in detail, a code network is presented (Figure 1) as an overall summary of the main categories and their interrelationships. Subsequently, for each thematic category, a specific schematic representation is provided (Figure 2, Figure 3, Figure 4 and Figure 5) to visually summarise its subcategories and associated codes. All quotations are coded to preserve participant anonymity. The coding indicates whether the respondent is a father (F) or a mother (M), and the accompanying number corresponds to the participant’s interview, allowing cross-reference with the child’s data in Table 1 and Table 2. As one father and nine mothers participated in the study, the term “mothers” is used hereafter to refer to participants collectively, with “father” specified only when highlighting characteristics unique to that participant.

### 3.1. Family Emotional Experiences of Childhood Fever

This category (Figure 2) encompasses the narratives in which mothers describe their experiences with previous episodes of childhood fever. The data are organized into two subcategories: (1) the perceived impact of fever on family dynamics, and (2) mothers’ interpretations of the physical effects of fever on their children.

#### 3.1.1. Impact of Childhood Fever on the Family

The subcategory of family impact is further divided into three dimensions: the maternal experience, the emotional responses triggered by the febrile episode, and the ways in which it disrupts or alters family organization and routines.

##### Mothers’ Perceptions of Childhood Fever

Previous experiences with childhood fever contribute to the development of what mothers describe as maternal intuition, an instinctive ability to recognize signs of severity and determine appropriate actions based on subtle behavioral or physical cues in their child. This intuition is frequently referenced in their narratives as a guiding element in fever management. Mothers with more than one child often highlight that febrile episodes can vary significantly between children, highlighting the individualized nature of each experience.

Mothers ultimately acquire knowledge and confidence in managing fever through lived experience; however, they also recognize that each child may respond differently and require distinct approaches. As one mother explained:

*When his sisters had a fever, I gave them the paracetamol shot. However, he is different. His body reacts differently to fever.*

M1

*This highlights the individualized nature of fever experiences within the same family and the adaptive strategies mothers develop over time.*

*Mothers in this study emphasized that their knowledge about fever management is primarily rooted in personal experience rather than formal education. One participant explicitly noted: I have actually learned more as a mother than I did from my studies (pharmacy).*

M2

*For many, repeated exposure to febrile episodes, especially during early childhood, has contributed to this experiential learning. As one mother remarked: Since school started, we have had one virus after another, and fever has become a constant in our household.*

M3

*This learning process is closely tied to a form of maternal sensitivity or intuition, which mothers describe as the ability to detect subtle changes in their child’s behavior or physiology that signal the onset of fever. For example, a father shared: “The first thing is to know what your baby is like. And to tell you the truth, it is the mother who notices it first. I mean, he is a very active baby, and when he is not active, it means that something is wrong” (F1). Well, I know when his fever is going to break when his feet and hands get cold. That is how I know. Then I say, “Oops, this child is going to get a fever”.*

M8

##### Emotional Aspects of Fever Management

Although fever is a common occurrence in childhood, it is frequently perceived by mothers as a potential indicator of serious illness, triggering emotional alert mechanisms. The predominant emotions expressed in participants’ narratives include concern, insecurity, and uncertainty, particularly regarding the progression of the febrile episode and the adequacy of their caregiving decisions. Some mothers also described a state of emotional ambivalence, characterized by an outward appearance of calm while internally experiencing doubt and apprehension. This tension highlights the complex emotional landscape surrounding the management of childhood fever, even among experienced caregivers.

*I suppose there’s a compromise, but I tend to worry a lot. Let’s focus on reducing the fever; the main thing is for the child to be okay and the fever to come down.*

M2

*Me? Not good at all. I’m very worried. I always associate fever with something serious. When he was diagnosed with diabetes, the first symptom was a fever.*

M7

*I feel conflicted. It’s like: Am I handling this correctly? Is it okay not to focus too much on the fever? Or should I see a doctor just to be safe? On one side, I’m calm, but on the other, I’m questioning if I’m doing the right thing.*

M3

*I know that if the fever goes above 37.5 °C, I should give him an antipyretic, but I’m not sure if that’s the right decision.*

M2

##### Beyond the Symptom: Managing Family Dynamics During Childhood Fever

For most participating mothers, fever is not just a physical issue but also disrupts family routines, as their child’s absence from school often means, depending on social support, that they either cannot go to work or someone else must care for the child.

*When the child has a fever, their usual routine is disrupted: the child stays home from school while I work. This means my husband and I must juggle work or depend on grandparents or someone else to care for the child.*

M2

*Sometimes, I can’t go to work or leave the child with my mother-in-law so that I can work for a while.*

M5

*Don’t mind; he comes first. I try to leave him with a family member, like my mother, who fully supports me. However, if she’s not available, I request at least a vacation day.*

M6

*Plans, we might skip therapy, school, or the park. Everything gets rearranged.*

M8

The main difficulty is that you can’t do everything you have to; you often have to take a day off work.

F1

#### 3.1.2. Variability of Fever Experiences Among Children

This subcategory covers the accounts in which mothers describe their observations of physical changes in their children during a fever. Some indicate noticeable differences in skin and limb color, overall condition, or behavior. Participants view these changes as unique to their own child and recognize they may differ in others. This perception is strongly influenced by the child’s baseline health status and the presence of any chronic illness. When the child has trouble expressing discomfort verbally, mothers focus on other signs, like labored breathing or body stiffness.

*His feet and hands turn purple, he starts trembling, dark circles appear under his eyes, his skin becomes pale, and his lips turn purple.*

M1

*He starts feeling weak, loses his appetite, and begins to fade; it is like deep fatigue or exhaustion. When he has a fever, he is not himself.*

M4

*Absolutely. When she has a fever, she gets very down, doesn’t want to eat, and stops playing like she normally does.*

M5

*Even with the same temperature, you touch him and he feels burning hot. His mood changes; he just lies there and stops being himself.*

M6

*Her breathing becomes very fast when her fever rises. Moreover, since she can’t express herself verbally, she stretches out and becomes very rigid; that’s how she shows it.*

M8

### 3.2. Maternal Decision-Making in Response to Fever: Balancing Rational, Emotional, and Cultural Factors

This second category (Figure 3) encompasses the discourses that refer to, or can be interpreted as, the factors that influence or shape the actions mothers take when their child has a fever. It includes the subcategories of knowledge, fear, social influence, and cultural influence.

#### 3.2.1. Mothers’ Knowledge About Childhood Fever

Based on their accounts, mothers’ knowledge has been categorized into three areas: pharmacological treatment, understanding fever as part of the body’s defense system vs. a potential danger, and monitoring for signs and symptoms.

##### Diverging Perspectives on Antipyretic Treatment for Childhood Fever

Mothers share varying views on medications, influenced by their understanding of antipyretics. Some consider them harmless tools to reduce the risks of rising temperatures, while others acknowledge potential side effects, believing these can be minimized through moderate use. An emerging concern is the unnecessary overuse of medication without a clear need. The use of antipyretics appears as an immediate reaction by mothers to childhood fever. They are primarily used to reduce temperature, though some mothers also administer them to ease the child’s general discomfort. However, one mother with a health background cautions against overuse, even for mild symptoms, highlighting a tendency toward automatic administration without fully evaluating the child’s condition.

*As long as you don’t overuse (antipyretics), they’re not harmful. In the end, the medication helps because I have a fever; I believe an uncontrolled high fever can be dangerous, I think.*

M1

*Yes, they’re used quite a bit, especially paracetamol or ibuprofen, not just for fever. For minor pain, we give a little paracetamol or something to help with sleep, which can mask symptoms. We often medicate unnecessarily.*

M2

*Ibuprofen does have many side effects, and we don’t fully know all of them. Reading the package insert can be scary. Paracetamol, however, is generally better tolerated regarding side effects.*

M3

*No, I don’t think so. These drugs might have some effects if used very regularly, but occasional use is fine. Alternating them is acceptable.*

M7

##### Dual Perception of Fever: Natural Defense vs. Potential Danger

Participants’ accounts reveal a conflict in their understanding of fever, between viewing it as a defense mechanism and fearing the harm a high temperature might cause the child. Some mention febrile convulsions as a possible risk linked to the risk of high temperature.

*Fever is an increase in body temperature caused by a viral or bacterial infection, acting as the body’s natural defense mechanism.*

M3

*For me, fever is when the body reacts to something external, signaling: “Hey, I’m fighting off something foreign or strange.”*

M6

*Dangerous? I don’t think so. Unless it’s very high level and causes severe discomfort or exhaustion. Otherwise, usually not.*

M3

*I consider a fever of 38 °C dangerous because it can rise to 39 °C, and at 40 °C, convulsions can occur.*

M5

*Yes, at some point, it can cause a seizure, but fever exists because the body is defending itself.*

M8

*If the temperature gets too low, it can be dangerous. That’s why knowing your child well and responding quickly is crucial.*

F1

##### Warning Signs of Fever as Identified by Mothers

The main symptoms mothers associate with the potential severity of an illness are the child’s body temperature, how effectively antipyretics lower the fever, and any signs of weakness. Only one mother indicated a different key indicator of seriousness during a fever episode, petechiae.

*You need to monitor fever spikes and how long they last. In this case, it went on for four days with very high peaks, and the fever wouldn’t come down.*

M1

*If it’s 38 °C, and I give medication but it doesn’t lower the fever, I’d go to the emergency room.*

M2

*I watch for petechiae. I’m constantly observing. Signs that go beyond just the fever.*

M3

*Apathy, giving paracetamol, and the fever still doesn’t drop; continued discomfort. Or if it lasts more than three days, that’s concerning.*

M3

*It is important to know your child well. When they seem fussy, don’t eat properly, or appear weak, that’s when we’re told to go to the emergency room right away.*

F1

#### 3.2.2. Fever and Fear: Emotional Influences on Maternal Care Decisions

Fear is a key factor influencing mothers’ actions and is clearly expressed in their accounts. They describe fear of fever itself, fear of rising temperatures, and fear of febrile convulsions. This fear often drives them to act quickly, frequently using antipyretics early, seeking emergency care, or closely monitoring their child for extended periods. In some cases, fear becomes overwhelming, leading to uncertainty and doubt about their own caregiving decisions.

*Yes, I’m afraid of fever.*

M1

*I try to focus on the discomfort, but it isn’t very comforting.*

M3

*I’m afraid of fever. Yes.*

M6

*I don’t let it reach a high temperature. I know it means the body is fighting harder, but I’m still scared of it getting too high.*

M6

*Yes, I’m afraid, because it can cause seizures.*

M7

*I think a high fever could affect the brain.*

M8

#### 3.2.3. Inherited Knowledge and Social Pressure in Fever Management

The participants report that much of their influence comes from what they learned from their mothers, who significantly shape their decisions. They are also affected by their social environment, which commonly views any rise in temperature as inherently dangerous.

*Yeah, I do what my mother used to do with me.*

M1

*People around me usually say that fever is bad, warning: “Be careful, don’t let it rise, might have a seizure. Be careful, or he could have an attack.”*

*I follow what my mother tells me. Start with cold water compresses…. She’s helped me a lot, reminding me to watch my daughter’s face, her eyes….*

M4

*I have learned from life itself, from how our parents handled things, like using wet clothes or giving aspirin, not Ibuprofen. And also, from society in general, from friends.*

M6

#### 3.2.4. Cultural Differences in Maternal and Paternal Perspectives on Fever

Cultural differences are evident in how parents manage a child with a fever. Immigrant participants generally expressed greater fear of fever compared to others and were less likely to follow the typical healthcare process, often choosing to go directly to the hospital. Among these participants, there is a noticeable urgency in lowering the child’s temperature. Some mothers highlighted differences in care practices compared to their home countries, and one expressed heartfelt gratitude for the medical care their child received. Notably, one father in this group demonstrated clearly defined gender roles in caregiving.

*If she gets a fever today, I’ll be at the hospital the next day. I go home, then return to the hospital. They explained the same thing to me.*

M5

*I have always taken her straight to the hospital. I have never gone to the pediatrician at the health center. Always the hospital.*

M5

*At home, if I see the temperature is 38 °C, I give both paracetamol and ibuprofen, and apply cold compresses to the head to bring it down as quickly as possible.*

M7

*When we notice a fever, we go to the health center. If it’s the weekend, we go to the hospital so they can tell us whether to give Paracetamol. We prefer the doctors to decide rather than give anything ourselves.*

F1

*Yes, I go to the health center. However, honestly, it feels like a waste of time if the fever hasn’t lasted three days straight; they don’t do anything.*

M7

*Back in my country, my mother would cover us up so we’d sweat it out; the more you sweat, the more the fever would disappear.*

M7

*The doctors here are amazing; they take great care of him, and everyone has love and support.*

F1

*As a father, your role begins with comforting the mother, as she tends to worry more. Women know the child better and notice things sooner.*

F1

### 3.3. Maternal Practices in Response to Fever: Balancing Home Care and Healthcare Access

After organizing the guiding factors in the previous category, this category (Figure 4) focuses on the actual decisions and actions mothers take when their child develops a fever. It is structured into two subcategories: care and seeking medical attention.

#### 3.3.1. Mothers’ Approaches to Managing Childhood Fever

Mothers’ care practices have been organized based on their objectives, i.e., whether the aim is to reduce the child’s discomfort or lower the temperature. This also includes the sequence they follow among the various care options they describe, as well as how and when they decide to seek medical attention.

##### The Role of Maternal Beliefs in Defining Care Objectives During Fever

Participating mothers approach caring for a febrile child with a clear intention, which varies between relieving the child’s discomfort and lowering the fever. Intentions are shaped by their prior beliefs, whether they view fever as part of the body’s defense system or as a potentially dangerous condition, as outlined in the previous subcategory.

*To lower the fever and get the child active again.*

M1

*To improve her well-being, to help her feel better and check if she’s in pain. The antipyretic helps with pain too, especially since she can’t always say if something hurts, like a headache.*

M3

*(What effect do you look for when you administer the antipyretic?) Yes, to reduce the temperature.*

M6

*I was told to give her the medication if her fever got too high.*

M2

*I suppose that’s what they told me to give it if her fever rises too much.*

M2

##### Personalised Care Strategies for Managing Fever

To reach their goal, whether it is easing discomfort, lowering the temperature, or identifying the cause, mothers follow a sequence of care options, starting with home care and, if needed, progressing to medical attention. This approach is tailored to each child’s specific needs, particularly in cases involving chronic conditions, where parents tend to follow a more structured and systematic series of actions.

*If they have a temperature of 37.5 °C and seem unwell, I give them paracetamol or ibuprofen to help them feel better.*

M2

*If the fever is high, I don’t cover them up too much; I keep them more uncovered so the temperature doesn’t rise further.*

M2

*I take off his clothes if he has a fever of around 37.5 °C; usually, that brings it down, along with a few other things. I might wet his face with a cold cloth, and if I see the fever rising, I give him an antipyretic, usually Paracetamol.*

M3

*No stress, no panic. We give her ibuprofen to lower her fever, and then she goes to the hospital, because in her case, staying at home with a fever is not safe. She needs IV fluids, and most importantly, we’re concerned about finding out what’s causing the fever.*

M4

*I’ve used cold compresses on her. We’ve also given her a bath and checked her temperature to ensure it drops and stays stable.*

M9

#### 3.3.2. Choosing Healthcare Services and Delegating Care to Professionals

Seeking professional care is shaped by the reasons behind choosing one health center over another, and in some cases, by the delegation of responsibility for managing the child’s fever to healthcare providers.

##### Duty and Decision: Tensions Between Health System Knowledge and Maternal Practice

When choosing a health center, participating mothers and fathers decide between primary care, hospital care, and, in some cases, private healthcare. Although they are aware that primary care should be the first option, factors such as the absence of paediatricians at their local centre or a perception of limited expertise often lead them to go directly to hospital emergency rooms. Mothers with private insurance report turning to public hospital emergency services when dissatisfied with private care.

*Initially, I visit the health centre, but if I don’t receive a satisfactory answer, I proceed to the hospital.*

M7

*I prefer to start at the health center because his pediatrician, who’s cared for him since birth, is there. I am lucky at my health center, they always communicate well and explain things clearly.*

M1

*They do fewer tests at the health centre, and sometimes they prescribe antibiotics for what might be a viral infection. I don’t like that, we could be treating something that isn’t even there.*

M2

*(Primary care impression) Very bad. Really bad. Not because of the fever itself, but because they leave you feeling alone, like you have to fight for your child’s well-being by yourself.*

M3

*I try to use the health center, but there’s often no pediatrician available, especially in urgent situations. Ideally, I’d trust them, but I don’t. You try to book an appointment, but there is nothing available for two weeks. So I end up going straight to the hospital.*

M3

*For me, it’s clear that I prefer a private doctor for minor issues like colds or gastroenteritis, as I don’t like the crowded environment of public hospitals. However, for serious health issues, I prefer public healthcare.*

M6

##### Professional Endorsement and Trust in Febrile Care Practices

Based on participants’ discussions, it appears that some mothers, when visiting emergency rooms, either at hospitals or primary care centres, aim to shift part of their child’s care to healthcare professionals. Even for familiar tasks like giving antipyretics, they often wait for professional confirmation, leading to frequent visits to these services.

*If he starts running a fever, I’ll give him some ibuprofen and then take him to the paediatrician, saying, “Look, I’ve given him ibuprofen.” I explain what I have done. I don’t give him anything without telling them. They tell me whether to keep giving ibuprofen or switch to antibiotics.*

M1

*I’m not the one who knows what to do about my son’s condition.*

M6

*We always rushed to the hospital. I would go, and they would tell me the same thing: it was a virus or a respiratory infection. My first instinct was always to get to the hospital quickly.*

M5

*The first thing I do is take him to the emergency room. I don’t give medication just for the sake of it. Sometimes they ask, “Did you give him Ibuprofen?” I say, “No, I wanted you to come, so you could see how bad he was.”*

M6

### 3.4. Mothers’ Perceptions of the Professional Care Relationship: Expectations, Experiences, and Learning

Finally, this category (Figure 5) encompasses discourses in which mothers describe their relationships with healthcare professionals during visits for fever. These interactions are organized around their needs and expectations, their perceptions of the professionals, and the type of healthcare education they have received.

#### 3.4.1. Mothers’ Need for Education on Childhood Fever

Mothers clearly express a need for more education on managing childhood fever and consider primary care the ideal setting for this. They believe such training would be most effective if provided through workshops or during routine child health program visits. Due to the lack of response from the health system, some mothers have turned to alternatives like private courses, which have helped them expand their knowledge and feel more confident when dealing with febrile episodes. In this context, mothers report receiving two main recommendations from healthcare professionals: to wait at least three days to observe the fever’s progression before seeking medical attention, and to administer antipyretics to lower the temperature. However, this information is sometimes seen as insufficient or not well aligned with the family’s real concerns, prompting them to seek knowledge through other means actively.

*For example, during the first routine visit with the pediatrician, when they explain many things, I think it would be helpful if they also talked about fever.*

M2

*It would be useful to have workshops for parents, similar to postpartum classes, but focused on first aid for children, nutrition, and possibly around the child’s first birthday. I took a course on my own with a pediatrician about Baby-led weaning and first aid, which included fever.*

M3

*I would have liked them to explain something about fever in the prenatal classes, to tell us that it’s very common.*

M9

*They advised that if a child has a fever, the first step is to monitor its progression. Next, try removing their clothes, applying warm water to their forehead, and if that doesn’t help, administer paracetamol.*

M1

*They always tell me I need to wait three days before taking him to the emergency room.*

M5

*(At the health center. Did they explain anything to you?) No. And here at the hospital, they have always said that if it’s not 38 °C, it’s not a fever.*

M6

*They only told me that if she complains, I should give her paracetamol.*

M7

*They explained that if my daughter has another seizure, the first thing I should do is lie her on her side. They told me that if the seizure doesn’t stop after five or ten minutes, though I don’t intend to wait that long, I should give her the medication orally and then call the emergency number, although I don’t remember exactly which one.*

M7

*They told us we had to wait at least three days, because with just a fever and no other symptoms, it’s very difficult to take action.*

M2

#### 3.4.2. Maternal Perspectives on the Aims of Professional Fever Management

Mothers perceive that health professionals focus primarily on reducing body temperature when caring for children with fever. This view is reinforced by immediate actions seen in clinics or emergency rooms, like quickly giving antipyretics or frequently checking the temperature. In many cases, this focus on fever control creates an impression of efficiency and thoroughness, reassures some mothers, especially when paired with exams and diagnostic tests. However, other mothers feel ambivalent or even worried about these practices. The number of tests can be seen as a sign of seriousness or cause doubts about whether the situation is being overstated.

*Some doctors place great importance on fever, while others do not.*

M3

*The first thing the professionals do is give antipyretics.*

M5

*Doctors and nurses want to keep the fever low, prevent it from rising, and shorten its duration. Their goal is to reduce it as quickly as possible, which I agree with.*

M1

*At the hospital, the nurses only take her temperature, so they say she doesn’t have a fever. The other day it was 37.6 °C, and they said, “Well, it’s 37.6 °C. Later, they checked again, and it was down to 36 °C.”*

M2

*No, they never told me: “Stay calm because what matters most is that the child is well, not just that the fever goes down”. Instead, the message is: “Paracetamol first, ibuprofen if that doesn’t work, and alternate between the two.”*

M3

*I just finished with the girl in the hospital. We’ve been here for two days, and I wonder: Was all this necessary? Maybe yes, maybe not. What did they see that made them hospitalize her? Why couldn’t we handle it at home?*

M3

*What the doctors give me is peace of mind, but also doubt and uncertainty about the diagnosis and what the results will show.*

M7

*They want to lower her temperature and find out where the fever is coming from. Whether it is an infection or something else.*

M8

## 4. Discussion

The analyzed discourses aimed to explore families’ perceptions of childhood fever, the factors influencing their care decisions, and their experiences across various care settings. The discussion is organized into four sections, each corresponding to one of the main categories of this qualitative study.

### 4.1. Family Emotional Memories of Childhood Fever

The results of this qualitative study reveal that mothers develop a growing intuition through experience to recognize early signs of fever in their children, aligning with the findings of Morris et al. [19] and Franklin et al. [43]. This intuition enables caregivers to personally interpret their child’s physical and behavioral changes, like skin color, discomfort, loss of appetite, or activity, even before fever is objectively confirmed. As Göbekli and Güney [44] highlight, the signs that most concern caregivers are not always biomedical, but those that disrupt the child’s usual behavior, validating parental experience as a legitimate diagnostic tool.

From this perspective, the ethnomethodological approach used in this study [36] was essential in revealing how mothers develop and validate this knowledge through everyday life. By examining often overlooked actions and discourses, like touching the child, interpreting their gaze, or anticipating behavioral changes, it was possible to understand how these practices are structured, give meaning, and guide child care beyond formal biomedical knowledge. In line with Harper’s ethnomethodological framework, these practical ways of “reading” and acting in daily life can be understood as members’ methods through which families construct and maintain social order around childhood illness [36].

The observational skills described by participants improve over time and rely heavily on personalized knowledge of the child. For example, several mothers indicate that they know “he is going to get a fever” because they notice cold extremities or changes in activity level. These accounts support Kuijpers et al. [5], who highlight that home-based clinical assessments depend on a mother’s emotional and physical reading of the child, developed through experience.

Participants also highlight that each child experiences fever uniquely, requiring them to adjust their observation and care approaches. This acknowledgment of individual differences moves away from standardized protocols and aligns with Göbekli and Güney [44], who stress that parental knowledge is not universal but shaped by medical history, age, the child’s expressiveness, and past experiences.

Another important aspect in the reports is the emotional burden of childhood fever. Mothers describe feelings of anxiety, fear, conflict, and insecurity during febrile episodes, particularly when the cause is unclear or initial treatments fail. Franklin et al. [43] argue that clinical judgment at home is not solely rational, but deeply shaped by emotions like guilt, fear of judgment, and uncertainty. These emotions are particularly strong in new mothers or those with children who have a negative medical history, such as febrile seizures or chronic illness. In our study, one mother whose child developed diabetes during a febrile episode shared that fever has since become a signal of increased alert, intensifying her emotional reaction.

The persistence of widespread misconceptions amplifies this emotional burden. In line with findings from Chulapornsiri et al. [45], where 68% of caregivers believe that fever can lead to brain damage or death, our participants express a clear fear of serious complications, even when they recognize fever as a natural defense mechanism. This tension between biomedical knowledge and emotional response is well documented [2] and leads to an ambivalent responses: while some mothers acknowledge that “fever is a sign the body is defending itself”, they still act swiftly and preventively to stop it from “getting too high”.

Another notable finding is the impact of fever on family organization. Mothers report that a febrile episode disrupts daily routine: it keeps the child from attending school, forces adjustments to work schedules, and often requires support from the family network, especially grandparents, to balance caregiving with other responsibilities. This phenomenon, as documented by Villarejo-Rodríguez and Rodríguez-Martín [46], highlights the structural impact of domestic care in cases of mild childhood illness, where women largely continue to bear the primary caregiving responsibilities.

The work dimension stands out as particularly significant. In the interviews, working mothers indicate having to request leave or use vacation days to care for their children, creating tension in both their professional and personal lives. This reality, also noted by Göbekli and Güney [44], shows how childhood fever, though clinically minor, can have major impacts on families, exposing a dis-connect between caregiving needs and the structural conditions for achieving work–life balance. From Bolívar’s perspective, these tensions can be read as the result of underlying attitudinal and normative patterns that shape how care, responsibility and work–family balance are socially distributed and perceived [37].

### 4.2. Maternal Decision-Making in Response to Fever: A Balance of Rational, Emotional, and Cultural Factors

This qualitative study found that a combination of emotional, cognitive and social factors strongly shapes maternal decisions during a febrile episode. This aligns with the findings of Villarejo-Rodríguez and Rodríguez-Martín [47], who argue that fever interpretation is not solely based on biomedical knowledge, which may resemble Freire’s concept of banking education [38], but is also informed by past experiences, life context, and the support network available. Similarly, Thompson et al. [8] highlight that decision-making around fever is a dynamic process influenced by intense emotions, incomplete knowledge, and the pressures of the family environment. In Freirean terms, much of the information that families receive about fever appears to be transmitted in a unidirectional way, with limited space for dialogue and critical reflection, which may restrict the development of more autonomous and reflective caregiving practices [38].

In the findings of this study, many mothers expressed an ambivalent stance toward the use of antipyretics. While they are seen as effective tools, there is also awareness of the potential risk of overuse. This aligns with Pitoli et al. [10], who note that parents often alternate or combine antipyretics during persistent fever episodes, despite the risk of overdosage or adverse drug interactions. Chulapornsiri et al. [45] further demonstrates that high parental anxiety levels are linked to dosing errors, reinforcing the strong connection between emotional states and the quality of decision-making. From Bandura’s social learning perspective, these patterns reflect behaviours that are shaped and maintained by internal reinforcement: actions that temporarily reduce the child’s discomfort and parental anxiety are more likely to be repeated, even if they are not fully aligned with clinical recommendations [17].

Mothers’ fear of their children’s fever reflects a conflict between their rational understanding of fever as a natural defense mechanism and their emotional response to its potential risks, an early stage of reflective caregiving in everyday life [36]. This “feverphobia”, documented in several studies [30,43], is often marked by intensified concern, even among trained caregivers, and is largely driven by fears of seizures or neurological damage, leading to anticipatory and defensive behaviors [17]. Bandura’s theory helps explain how stories of adverse events, whether experienced directly or heard from others, can act as powerful vicarious experiences that heighten fear and lower self-efficacy, thus favouring more defensive and risk-averse decision-making [17].

The social environment intensifies the perception of fever as a threat. In this study, pressure from other adults and shared experience with peers reinforce the belief that fever is dangerous. This finding aligns with Franklin et al. [43], who highlight the influence of social capital in shaping behavior in response to fever. When such support is lacking, reliance on emergency services tends to rise, a pattern also noted by Kassisse et al. [48], who link elevated levels of feverphobia to increased hospital utilization.

Regarding the symptoms that guide action, mothers in the study prioritize body temperature and the child’s weakness as key indicators of severity. This aligns with the findings of Morris et al. [19], who observed that a temperature perceived as “high” often prompts medical or pharmacological intervention. Intergenerational legacy is another important factor. Mothers’ reports show they often replicate care practices learned from their own mothers, such as using cold clothes or administering medications early. Villarejo-Rodríguez and Rodríguez-Martín [46] note that these inherited practices hold significant influence and are frequently prioritized over professional advice. However, this can create tension when family beliefs conflict with current health guidelines [8]. In terms of Bandura’s social learning theory, these findings reflect observational learning processes, in which maternal figures act as models whose practices are observed, internalized and later reproduced in the care of the next generation [17].

Immigration status and experience with the healthcare system also appear as influential factors. Immigrant mothers in the study exhibited greater concern and less trust in primary care. While Kassisse et al. [48] did not find a significant link between immigration status and fever phobia, the qualitative findings suggest that factors like gratitude toward the host healthcare system or past experiences in their countries of origin may shape behaviors. This perspective helps explain the ethnographic analysis of discourses.

Fear was also identified as a key motivator of action. This fear spans all educational levels, as demonstrated by Franklin et al. [43], and can lead to both early medical intervention and urgent medical consultations.

Finally, structural factors like educational level and gender also influence the response. Villarejo-Rodríguez and Rodríguez-Martín [46] highlighted the enduring narrative that credits mothers with superior disease detection skills based on “maternal instinct”. However, Shimony-Kanat et al. [18] found that six months after birth, many mothers see their partners as the main support in fever care, suggesting a possible move toward greater shared responsibility. From Bolívar’s perspective, these narratives and expectations can be seen as part of an attitudinal content that is transmitted and reinforced in families and healthcare institutions, shaping how caregiving roles are defined and distributed [37].

### 4.3. Maternal Practices in Managing Fever: Between Home Care and Healthcare Access

The results of this study indicate that mothers’ decisions and actions during childhood fever episodes are guided by practical objectives: alleviating the child’s discomfort, managing body temperature, and identifying the cause of the fever. This goal-oriented approach has also been documented by Villarejo-Rodríguez and Rodríguez-Martín [46] who observe that caregivers combine pharmacological and non-pharmacological strategies based on the perceived severity and progression of the illness. When these goals are unclear, it can result in disorganized and inconsistent behavior, as highlighted by Bolivar [37].

In this study, mothers exhibit continuous vigilance in response to fever, with body temperature serving as the primary trigger for both clinical and household decisions. This is reflected in frequent temperature checks, repeated antipyretic administration every four hours, and a series of interventions, such as undressing the child, applying cold packs, or giving warm baths.

These practices align with the findings of Gulcan and Sahiner [30] who reported that 41.3% of mothers used warm baths, 32.2% removed clothing, and 89.7% sought hospital care if the fever persisted. Similarly, Göbekli and Güney [44] observed immediate antipyretic use in 94.1% of caregivers and nighttime temperature monitoring in 59.4% of cases. Different strategies were also observed in deciding when to seek healthcare: 33.5% did so after the initial onset of fever, while 45.9% waited at least one day. This reflects the variability reported by the participants in the present study.

As Thompson et al. [14] note, initial autonomy in managing fever often diminishes when symptoms persist, leading to reduced confidence and a greater reliance on external support. This progression can be interpreted as behavior shaped by internal reinforcement, consistent with Bandura’s theory [17], where temperature acts as the behavioral stimulus. From this viewpoint, both symptom relief and decreased anxiety operate as reinforcers that consolidate certain patterns of action, even when they may not be clinically necessary [17].

In this regard, pediatricians in a qualitative study observed that families feel the need to “do something” when their child has a fever, such as attempting to reduce the temperature. They acknowledge that this response can be valid if it helps ease parental anxiety. However, they also criticize the frequent use of antipyretics as a quick solution [49].

Regarding the care itinerary, the mothers interviewed in this study often prioritize hospital care over primary care, even though they initially express a preference for their primary care pediatrician. This trend is also noted by Villarejo-Rodríguez and Rodríguez-Martín [47], who highlight factors such as limited appointment availability, the absence of a consistent pediatrician, and low confidence in the health center’s ability to resolve issues as key reasons families turn to emergency services. This decision is further shaped by emotional and social influences, including external pressure and the perceived need for an immediate physical assessment [43].

Furthermore, the results indicate that some mothers interchange between the public and private healthcare systems based on the perceived severity of their child’s condition. This strategy, also described by Morris et al. [19], reflects a pragmatic approach that prioritizes immediacy and diagnostic quality. In this context, the public system is often viewed as the final and definitive option when the private system falls short of expectations.

A key finding in this study is the tendency among mothers to seek professional validation before initiating or confirming certain actions. Even when they know when to administer antipyretics, they often prefer to have that decision endorsed by a healthcare provider. Franklin et al. [43] also emphasize this behavior, noting the important emotional role of clinical encounters in sharing responsibility and reducing the burden of decision-making.

Visiting the emergency room provides not only clinical assessment but also legitimacy and emotional support. Qualitative studies of doctors and nurses in the same setting identify these attitudes as a delegation of childcare responsibility to professionals [49,50]. The use of the thermometer reading as a strong justification for seeking consultation, observed in this study, has also been noted by Morris et al. [19]. Body temperature serves as a symbolic figure that connects subjective concern with objective action. This measurement not only directs care at home but also legitimizes the decision to seek medical attention in the eyes of others. From Bolívar’s perspective, the centrality granted to the temperature reading can be interpreted as part of a “hidden curriculum”, in which certain conceptual, procedural and attitudinal contents about fever (what is important, what must be controlled, what is dangerous) are implicitly transmitted and internalized by families through their interactions with professionals [37].

Finally, it is important to highlight the tension mothers face between acting promptly and waiting, or between intervening and avoiding over-medicalizing. This dilemma appears throughout the discourses and is linked to the participants’ confidence in their own judgment. Urbane et al. [3] note that parental self-efficacy is not fixed but fluctuates, often diminishing as the clinical condition persists or new uncontrolled symptoms emerge, creating a need for professional reassurance. Bandura’s work on self-efficacy and outcome expectations provides a coherent framework for understanding these fluctuations and the resulting search for external validation [17].

### 4.4. Mothers’ Perceptions of the Care Relationship: Expectations, Experiences, and Lessons Learned

The results of this study reveal an ambivalent relationship between mothers and health professionals in managing childhood fever, consistent with Franklin et al. [43], who highlight caregivers’ emotional pressure and their wish for their intuition and knowledge of the child’s condition to be acknowledged. Participants view the local pediatrician positively when care is continuous and communication is clear, but they also report a lack of explicit health education during routine visits. This perception aligns with Shimony-Kanat et al. [18], who found that only 62% of mothers had received formal information about fever six months postpartum.

Mothers specifically suggested the need for structured training, recommending workshops or group sessions within the public health system. This suggestion aligns with the findings of Tavan et al. [51] and Toksoz & Acikgoz [15], who showed that targeted educational interventions notably enhance caregivers’ knowledge and practices. In Freire’s terms, these demands can be understood as a call to move beyond a “banking education” model and towards more dialogical, participatory forms of health education in which families’ experiences and questions are actively incorporated [38].

Mothers observe that professionals tend to focus on reducing fever with antipyretics, which causes insecurity if not paired with a clear explanation. Franklin et al. [43] report that generic diagnoses like “viral illness” leave parents unsatisfied, failing to fulfill their need for understanding and control. The discrepancy between clinical guidelines and medical practice, such as emphasizing the thermometer reading over the child’s overall condition, has also been highlighted by Thompson, Nesari et al. [8] and helps perpetuate feverphobia.

Diagnostic testing generates conflicting perceptions: while some mothers view it as a safety measure, others associate it with unnecessary severity. This aligns with Leigh et al. [52], who noted that both the pain from testing and prolonged waiting times are significant sources of anxiety for caregivers. Likewise, Göbekli & Güney [44] found that 83.3% of mothers experience anxiety during febrile episodes, with 34.3% reporting fear, highlighting the critical need for emotional support during medical care.

Trust in healthcare professionals is also influenced by accessibility and the quality of relationships. Mothers expressed frustration with the shortage of pediatricians, challenges in securing appointments, and the occasionally impersonal attitude of some professionals. Villarejo-Rodríguez & Rodríguez-Martín [47] highlight that these barriers often lead families to prefer emergency departments, which are perceived as offering greater diagnostic capacity and emotional support. Supporting this, Chulapornsiri et al. [45] found that 70% of caregivers expected an accurate diagnosis, 67.4% anticipated the administration of antipyretics, and 52% expected antibiotic prescription.

Inconsistent clinical criteria among professionals and fragmented care further weaken trust in the healthcare system. Franklin et al. [43] warn that such inconsistency worsens caregivers’ uncertainty, who seek clear, accessible, and empathetic care. In this context, Morris et al. [19] emphasize that specialized services, such as pediatric emergency departments, provide not only a more comprehensive clinical approach but also a greater degree of validation of the parental role.

Nurses participating in a qualitative study conducted in the same setting expressed that implementing daily management recommendations for childhood fever would be very challenging, as it could lead to conflicts with families and increased anxiety when the child’s temperature does not decrease [50].

However, the underutilization of nurses as a source of guidance is also evident in the data. Only 32.4% of mothers considered nurses a primary source of information, compared to 69.4% who preferred physicians [44]. This disparity highlights the need to revalue the strengthening educational and supportive role of nurses in assisting families during febrile episodes. From Bolívar’s and Freire’s perspectives, this underuse of nursing as an educational resource suggests that the potential for a more dialogical, reflective and attitude-transforming pedagogy in clinical practice is not being fully realised [37,38].

#### Methodological Limitations and Scope of Applicability

This study presents several methodological limitations that should be interpreted in light of the logic and quality criteria of qualitative research. First, access to participants was mediated by intermediaries or key informants, which may have influenced the profile of the mothers and fathers who agreed to take part. The sample was purposive and context-dependent; therefore, the findings are not intended to be statistically generalisable, but rather to provide analytical insight and support the transferability of the results to settings with similar sociocultural and organisational characteristics.

Second, although the analysis was based on an ethnomethodological approach and informed by a plural theoretical framework (Harper, Bolívar, Freire and Bandura), fieldwork was conducted over a limited period and did not include prolonged ethnographic immersion, as proposed in some classical ethnographic designs. In this study, data were generated through in-depth interviews and the principal investigator’s field diary. While this strategy limits continuous, direct observation of families’ everyday lives, the combination of detailed narratives, contextual information and reflexive notes contributes to strengthening the credibility and interpretive depth of the findings.

Third, there is a relative scarcity of qualitative studies that integrate comparable theoretical frameworks in the analysis of childhood fever. This restricted the possibilities for direct comparison with previous research grounded in ethnomethodology, critical pedagogy or social learning theory. At the same time, this theoretical positioning allows the present work to be viewed as an exploratory contribution that may inform and guide future qualitative studies on childhood fever in other contexts.

Despite these limitations, the findings have practical implications for healthcare professionals, particularly in paediatric nursing and primary care. The results highlight the need to recognise and legitimise the experiential knowledge that mothers and fathers develop about their children as a relevant source of information in clinical assessment; to address persistent misconceptions and “fever phobia” through more dialogical and reflective forms of health education, rather than exclusively through unidirectional information-giving; and to review professional messages that implicitly prioritise numerical temperature over the child’s overall condition, as these may inadvertently reinforce defensive responses and excessive medicalisation. In addition, the study underscores the potential of nurses as key educational and supportive figures for families during febrile episodes.

In summary, the scope of applicability of these findings does not lie in their numerical representativeness, but in the extent to which clinicians, nurses and policymakers identify similarities between the contexts described and their own. The detailed description of mothers’ and fathers’ discourses, together with the explicit presentation of the theoretical frameworks used, is intended to help readers assess the transferability and practical relevance of these results for their specific settings and clinical practice.

## 5. Conclusions

This qualitative study reveals that childhood fever is not merely a clinical issue but a profoundly emotional and relational experience for caregivers, particularly mothers. Decisions surrounding fever are guided by a practical logic that interweaves prior knowledge, fears, cultural beliefs, and lived experience. Rather than relying solely on intuition, maternal management of fever reflects complex adaptive strategies designed to safeguard the child while also seeking validation and support from the healthcare system. The ethnomethodological approach has enabled the recognition of how mothers interpret and assign meaning to everyday practices that often go unnoticed due to their routine nature. By framing these actions as social phenomena, the underlying meanings that shape home-based childcare are brought to light. In this context, the role of healthcare professionals becomes central, not only for their technical expertise but also for their capacity to communicate, support, and validate parental experience. The findings highlight the importance of integrating this perspective into clinical practices and the development of educational interventions, fostering more empathetic, responsive, and context-sensitive support that aligns with the emotional and social realities of home-based childcare.

## Figures and Tables

**Table 1:** Socio-demographic data of parents.

Code	Age	Sex	Nº of Children	Educational Level	Perceived Support	Home Country
M1	36	Female	3	Secondary school	High	Spain
M2	35	Female	2	University degree	Medium	Spain
M3	32	Female	1	University degree	High	Spain
M4	52	Female	2	Secondary school	High	Spain
M5	28	Female	1	Technical education	High	Cuba
M6	46	Female	1	University degree	High	Spain
M7	42	Female	2	Secondary school	Low	Ecuador
M8	36	Female	1	Technical education	Medium	Spain
M9	26	Female	1	Technical education	Medium	Bolivia
P1	40	Male	1	Primary school	Low	Nigeria

**Table 2:** Children’s data provided by parents.

Code	Age	Sex	Chronic Illness	Reason for Hospitalization	History of Febrile Seizures
M1	3 years	Male	No	Fever/Rinhovirus	No
M2	4 years	Male	No	Bacterial skin infection	No
M3	21 months	Female	No	Fever	No
M4	12 years	Female	Yes (LCHAD)	Fever	No
M5	4 years	Female	No	Fever	No
M6	9 years	Male	No (Crisis de ausencia)	Gastroenteritis	No
M7	10 years	Male	Yes (Diabetes)	Fever/skin eruptions	No
M8	3 years	Female	Yes (Cerebral palsy)	Pneumonia	Yes
M9	5 months	Female	No	Bronchiolitis	No
P1	7 months	Male	No	Respiratory virus	No

## Data Availability

The raw data supporting the conclusions of this article will be made available by the authors on request. The data are not publicly available due to ethical restrictions and the need to protect participants’ confidentiality.

## Supplementary Materials

The following supporting information can be downloaded at: https://www.mdpi.com/article/10.3390/children12121584/s1, Standards for Reporting Qualitative Research (SRQR) guidelines. Reference [53] are cited in Supplementary Material.
